# Development of an artificial intelligence-based computer-aided detection system for routine gastric biopsy diagnosis

**DOI:** 10.1016/j.jpi.2026.100654

**Published:** 2026-03-19

**Authors:** Daiki Taniyama, Kazuya Kuraoka, Akihisa Saito, Aya Kido, Ayaka Shirai, Rie Yamamoto, Masayuki Mano, Norihiro Teramoto, Shiro Miura, Morihisa Akagi, Norihiko Katayama, Tomonori Kawasaki, Chika Nakajima, Susumu Kikuchi, Kiyomi Taniyama

**Affiliations:** a Developmental Therapeutics Branch, Laboratory of Molecular Pharmacology, Center for Cancer Research, National Cancer Institute, National Institutes of Health, Bethesda, MD, USA; b Department of Molecular Pathology, Graduate School of Biomedical and Health Sciences, Hiroshima University, Hiroshima, Japan; c Department of Diagnostic Pathology, National Hospital Organization Kure Medical Center and Chugoku Cancer Center, Kure, Japan; d Institute for Clinical Research, National Hospital Organization Kure Medical Center and Chugoku Cancer Center, Kure, Japan; e Department of Clinical Laboratory and Surgical Pathology, National Hospital Organization Osaka National Hospital, Osaka, Japan; f Department of Pathology, National Hospital Organization Shikoku Cancer Center, Matsuyama, Japan; g Department of Diagnostic Pathology, National Hospital Organization Nagasaki Medical Center, Omura, Japan; h Department of Internal Medicine, Hiroshima Memorial Hospital, Hiroshima, Japan; i Department of Internal Medicine, Kure Medical Association Hospital, Kure, Japan; j Department of Pathology, Saitama Medical University International Medical Center, Hidaka, Japan; k R&D Japan, Evident Corporation, Tokyo, Japan

**Keywords:** Gastric biopsy, Computer-aided detection system, Digital pathology, Artificial intelligence

## Abstract

To reflect real-world pathology practice, we developed an artificial intelligence-based pathological computer-aided detection system, trained on diverse epithelial and non-epithelial tumors for gastric biopsy specimens. A multicenter cohort comprising samples from six institutions was used for training and validated with an independent dataset from a seventh institution. We applied two distinct algorithms and three operational validity levels with optimized parameters to address the complexity of pathological diagnosis, reflecting routine diagnostic practice. Our system enabled the detection of malignant regions at low-magnification observation, aligning with the practical workflow of pathologists. In a reader study involving a limited number of test samples, the use of system assistance was associated with an improvement in diagnostic sensitivity. Further analysis revealed that samples with small and dispersed malignant foci had a higher rate of false-negative diagnoses, underscoring the potential of our system to improve diagnostic sensitivity. This study highlights the promise of integrating our system into real-world practice to aid pathologists in the routine diagnosis of gastric biopsy specimens.

## Introduction

Gastric cancer, the fifth most diagnosed malignancy globally, accounts for over 1 million new cases annually and ranks as the third leading cause of cancer-related deaths worldwide.^1^ In Japan, many cases of intramucosal or early gastric cancer are detected annually through biopsy of erosive gastric mucosa via esophago-gastroduodenal endoscopic examination. However, a significant number of biopsy specimens do not contain malignant cells. Based on our experience in Japan, the positive ratio (malignancy number/biopsy sample number) ranges from about 10–50% in several hospitals. Differentiation of these cases requires specialized knowledge and experience of board-certified pathologists through microscopic observation. The shortage of board-certified pathologists is an urgent issue in Japan, as well as globally.^2^^,^^3^ Microscopic observation is gradually shifting to whole-slide image (WSI) observation on monitors within computer-based digital pathology systems. Artificial intelligence (AI) is being integrated into these digital pathology systems for various organs.^4^^,^^5^ Implementing AI in digital pathology systems may alleviate the burden on board-certified pathologists by aiding in the diagnosis of gastric biopsy specimens.

We have developed an AI-based pathological computer-aided diagnosis (CAD) system specifically designed to reach a high-sensitive performance in gastric biopsy diagnosis using hematoxylin and eosin (H&E)-stained WSIs. Seven hospitals in Japan were enrolled for the training and validation of this system. In addition to epithelial tumors, mucosa-associated lymphoid tissue (MALT) lymphomas, diffuse large B-cell lymphomas (DLBCLs), and gastrointestinal stromal tumors (GISTs) were included to reflect real-world practice. We applied two distinct convolutional neural network (CNN) algorithms to account for the histopathological heterogeneity, with a particular focus on detecting signet ring cell carcinoma (Sig). To accommodate diagnostic perspectives among observers, we implemented a multi-region of interest (ROI) framework that facilitates pathologists in retrieving suspicious regions at low magnification and supports definitive diagnosis at high magnification. To evaluate the clinical utility of our implemented system, we conducted a reader study in which five board-certified pathologists, accredited by the Japanese Society of Pathology, interpreted gastric biopsy WSIs with and without systems assistance. We demonstrated that diagnostic sensitivity improved with system assistance, although the difference did not reach statistical significance owing to the limited number of cases. Additionally, further analysis revealed that samples with small and dispersed malignant foci were associated with a higher rate of false-negative diagnoses, both with and without system assistance. These findings may highlight the potential of our system to improve diagnostic sensitivity in the routine practice of pathology for gastric biopsy specimens.

## Material and methods

### Basic design of AI algorithms

To assist pathologists in detecting malignant regions from digital pathology images, the concept of our AI-based CAD is categorized as CADe.^6^ As the principle of AI, we use CNN methods, also known as deep learning.^7^ To account for the histopathological heterogeneity observed across distinct malignancies, we implemented two distinct CNN algorithms. For malignancies characterized by widespread involvement within the pathological specimens, such as adenocarcinoma, we employed pyramid scene parsing network^8^ (PSPNet) as a semantic segmentation approach. Conversely, to detect Sig, which is distinguished by single cell size, we utilized the YOLO^9^ algorithm as an object detection method.

**Supplementary Fig. S1:**
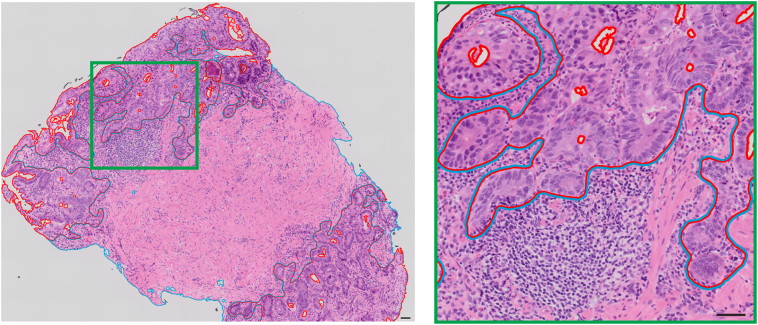
Example of annotation by pathologists. Three board-certified pathologists manually annotated malignant regions on the training images, which were subsequently used to ground-truth labels for training the CNN algorithms. (Red; Malignant, Blue; Benign, Black; Exclusion). CNN, convolutional neural network. Scale bar = 50 μm.

A training image set for the PSPNet was curated to include benign samples as well as comprehensive spectrum of malignant histologies,^10^ including papillary adenocarcinoma (Pap), tubular adenocarcinoma (Tub), poorly cohesive carcinoma (Por), and mucinous adenocarcinoma (Muc), MALT lymphomas, DLBCL, and GISTs. For YOLO, a distinct training set was constructed comprising images of Sig, alongside normal cell types-including Goblet cells that share morphological features reminiscent of Sigs, such as intracytoplasmic mucin accumulation and peripheral nuclear displacement. To construct the training dataset, WSIs were acquired from pathological glass slides using a slide scanner VS-M1 produced by Evident Corporation (Tokyo, Japan) at the magnification equivalent to 40×, which equates to a WSI pixel size of 0.23 μm on the slide. Three board-certified pathologists (DT, KK, KT) with specialized expertise in the long-term follow-up of gastric and colorectal adenomas over a period of 20 years or longer^11^^,^^12^ manually annotated malignant regions on all the training images, which were subsequently used to ground-truth labels for training the CNN algorithms (Supplementary Fig. S1). Following training, the PSPNet produces a pixel-wide malignancy probability map for each input image, with probability values ranging from 0 to 1, based on semantic segmentation. Notably, the annotated regions in PSPNet encompass both malignant areas and adjacent stromal areas. However, the inclusion of benign images containing stromal areas in the training dataset enables the network to learn reduced malignancy probabilities for stromal areas, thereby mitigating false-positive predictions. The trained YOLO outputs probability distributions for Sig in the form of rectangular bounding boxes centered on candidate cells, consistent with standard object detection methodology. The classification performance for distinguishing Sigs from Goblet cells was further enhanced through dedicated YOLO training.

We statistically evaluate the performance of the CNN algorithms based on receiver operating characteristics (ROC) analysis^13^ using an evaluation image set consisting of WSI samples. Ground truth was determined by microscopic examination of H&E-stained glass slides and, for challenging cases, by consensus among three board-certified pathologists, supplemented by WSI review on a monitor by the most experienced pathologist (KT). We draw a ROC curve by plotting the true-positive rate (TPR) and the false-positive rate (FPR) while scanning probability value between 0 and 1, where the true positive is defined when the maximum value of the malignant probability distribution for a malignant evaluation image exceeds the probability value, and the false positive when the maximum value for a benign evaluation image exceeds the probability value. We employed the area under curve (AUC) for the ROC curve as a statistical metric to evaluate the algorithm performance. Based on our CADe concept, we generated integrated ROC curve and corresponding AUC values by combining ROC analyses of both CNN algorithms. As a result of our evaluation, the AUC for the test image set described in the following section was calculated to be 0.971.

### Definition of CAD output using ROI to indicate malignant regions

**Fig. 1:**
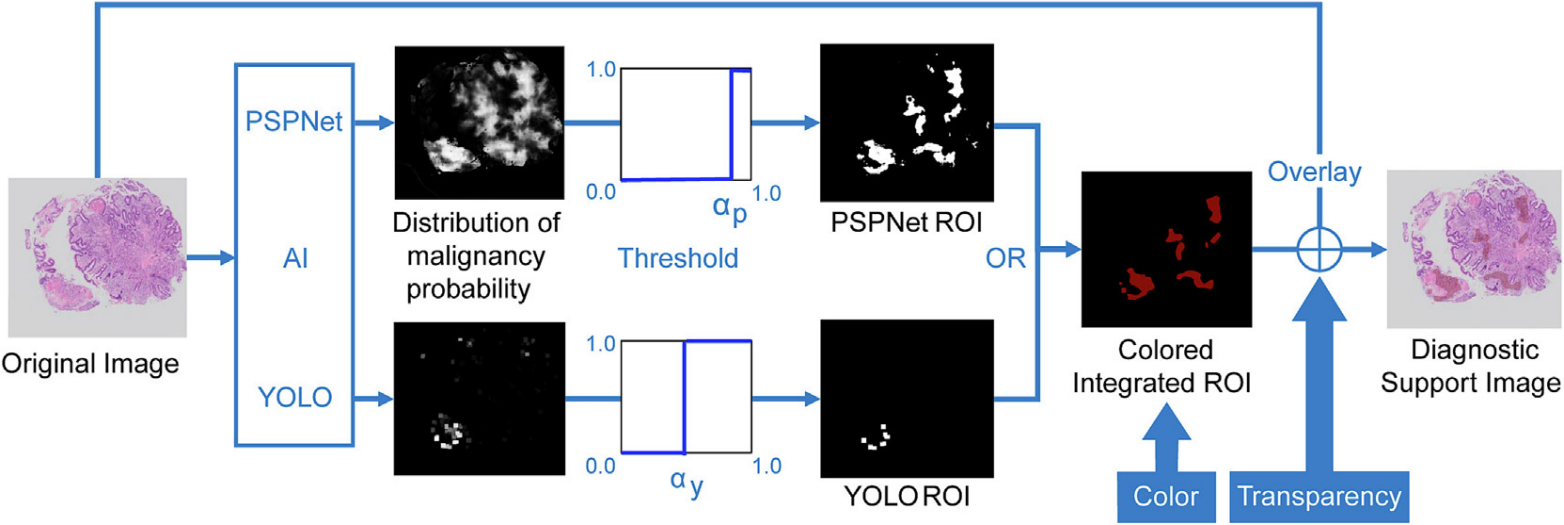
Schematic overview of the workflow for constructing a diagnostic support image. A binary region of interest (ROI) is extracted from the malignant probability distribution provided by the CNN algorithm, with values above a certain threshold. The ROI is overlaid onto the original pathological image with a dark red, 25% transparent highlight, enabling efficient diagnostic support through interpretable information. The integrated ROI is derived from a logical OR calculation of two ROIs obtained via PSPNet and YOLO. AI, artificial intelligence. (For interpretation of the references to color in this figure legend, the reader is referred to the web version of this article.)

We extract a binary image as ROI with values greater than a threshold from the malignant probability distribution provided by the CNN algorithm, which is suggested by AI to be highly suspicious for malignancy. We build a diagnostic support image as CAD information by overlaying the ROI onto the original pathological image with appropriate color and transparency. This ROI-based method effectively enables efficient diagnostic support by interpretable information for users, while maintaining low computational demands. Based on the CADe concept, we construct an integrated ROI through the logical OR calculation of two ROIs obtained by the PSPNet and YOLO. Fig. 1 presents a schematic overview of the workflow for constructing a diagnostic support image, in which a dark red overlay with 25% transparency was selected to highlight relevant regions.

### Design of diagnostic support tailored to observational intents

**Fig. 2:**
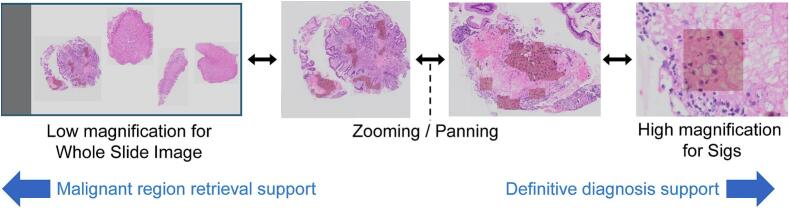
An AI-CAD design concept according to the image magnification. Our system mirrors the pathologists' workflow by initially identifying putative malignant regions at low magnification through architectural disruptions, and then examining cytological and structural atypia at high magnification for definitive diagnosis. It enables seamless magnification transitions and spatial navigation, facilitating the retrieval of suspicious regions at low magnification and supporting definitive diagnosis at high magnification. AI, artificial intelligence; CAD, computer-aided diagnosis; Sig, signet ring cell carcinoma.

Pathologists usually identify putative malignant regions by initially observing wide-field images at low magnification, focusing on architectural disruptions, followed by examination of cytological and structural atypia at high magnification to make a definitive diagnosis. This interpretive framework is recapitulated by digital platforms that enable seamless magnification transitions and spatial navigation. To recapitulate this diagnostic workflow, we developed an AI-CAD system designed to facilitate the retrieval of suspicious regions at low magnification and to support the definitive diagnosis at high magnification. The conceptual framework of the AI-CAD system, stratified by image magnification, is illustrated in Fig. 2.

### Dataset for training

Between January 2010 and March 2023, 13,109 samples with dataset of corresponding pathological diagnoses and clinical findings from 4154 patients were collected from 7 hospitals in Japan and submitted to the research secretariat, from which training samples were extracted. The training cohort was collected from six hospitals. The PSPNet training cohort included 951 WSIs, comprising 1925 samples. Among these samples, 1105 were benign and 820 were malignant, reflecting the spectrum of gastric specimens encountered in routine practice (Supplementary Table S1). For Sig detection, the YOLO training cohort consisted of 756 benign WSIs (1373 samples; 208,068 patches) and 60 malignant WSIs (93 samples; 1915 patches). Given that WSIs usually encompass multiple specimens (Fig. 2), each sample was treated as an independent unit of analysis.

## Results

### Optimization of the ROI threshold and provision of three distinct modes of CAD output

Given the heterogeneity of diagnostic perspectives among observers, ROI with a single threshold may fail to encompass the full spectrum of pathological features. To accommodate this variability, we implemented multi-ROI framework that enables selection of diagnostically relevant regions tailored to individual interpretive needs. To facilitate the retrieval of malignant regions at low magnification, ROI are broadly defined, enabling pathologists to readily identify suspicious areas during wide-field observation. Conversely, at high magnification, ROI are concentrated in regions with a high probability of malignancy, thereby assisting pathologists in making definitive diagnosis. Based on the ROC analysis, we optimize the characteristics of ROI by balancing TPR for malignancy and FPR for benign regions. A wide-spread ROI can be generated by lowering the threshold, thereby increasing TPR; however, this approach also increases FPR and results in inclusion of more false-positive malignant regions. Conversely, increasing the threshold decreases FPR and yields ROI that are concentrated in areas with higher probability of malignancy, thus reducing false positives.

Based on this concept, we designed three ROIs. The first ROI is derived from PSPNet outputs using a lower threshold, facilitating the retrieval of suspicious regions encompassing a wide spectrum of malignant types, and is primarily intended for low magnification observation. The second ROI combines the first ROI with regions identified by YOLO to enable the retrieval of Sig features, supporting their identification at high magnification. The third ROI is generated from PSPNet using a higher threshold, producing a more concentrated region to support definitive diagnosis at higher magnification. Diagnostic support images constructed using these ROIs are referred as OVL1, OVL2, and OVL3, respectively.

ROC analysis was performed using 211 benign and 324 malignant samples from the seventh hospital to optimize the ROI threshold. Classification criteria and number of samples used in the analysis are shown in Table 1. 306 G5 samples included 40 well differentiated Tub, 85 moderately differentiated Tub, 17 Pap, 13 solid type Por, 124 non-solid type Por, 15 Sig, 3 Muc, and 9 MALToma. Two G2 samples were suspicious for tubular adenoma with low-grade dysplasia, and three G3 were tubular adenoma with low-grade dysplasia. The 18 G4 samples comprised 11 suspicious for MALToma, 5 suspicious for DLBCL, 1 suspicious for moderately differentiated Tub, and 1 suspicious for non-solid type Por.

**Table 1:** Classification criteria and number of samples used for ROC analysis and evaluation.

Classification	Group	Definition	ROC analysis	Evaluation of CAD
Benign	G1	Benign	201	92
	G2	Suspicious for benign tumor	2	0
	G3	Benign tumor	8	7
Malignant	G4	Suspicious for malignancy	18	2
	G5	Malignancy	306	98

ROC, receiver operating characteristics; CAD, computer-aided diagnosis.

**Fig. 3:**
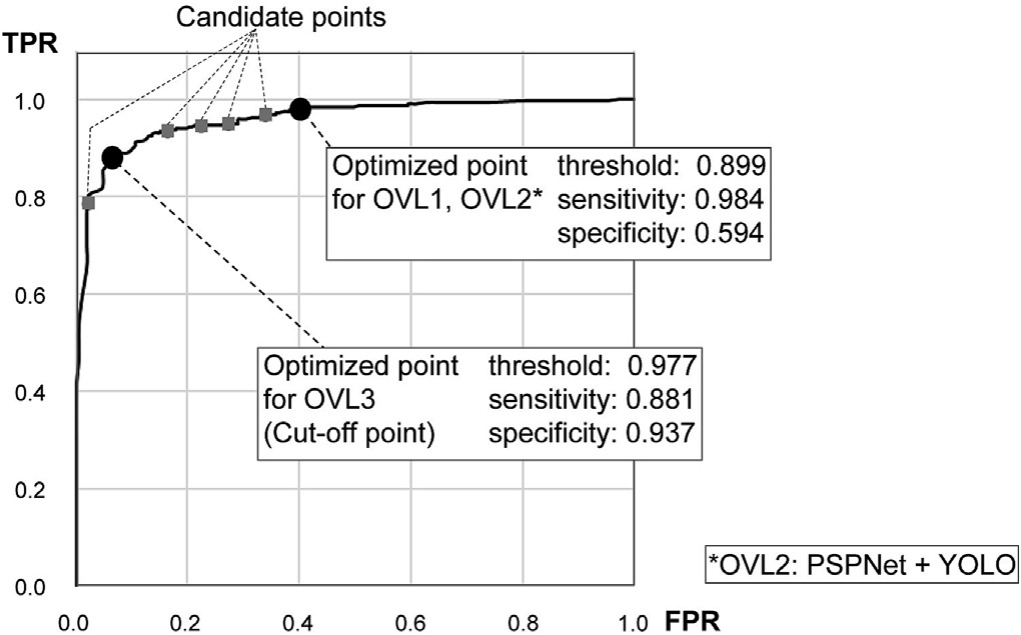
Receiver operating characteristic (ROC) curve for PSPNet. The curve shows the performance of PSPNet, with selected points used to construct OVLs. The designated cut-off points and several candidate points along the ROC curve are indicated. OVL1 is derived from PSPNet outputs using a lower threshold, facilitating the retrieval of suspicious regions across a wide spectrum of malignant types for low magnification observation. OVL2 combines OVL1 with regions identified by YOLO to support the identification of Sig features at high magnification. OVL3 is generated from PSPNet using a higher threshold, producing a more concentrated region to support definitive diagnosis at higher magnification. FPR, false-positive rate; TPR, true-positive rate.

We optimized the ROI thresholds for the OVLs referring to the ROC curve as follows. Threshold favoring higher TPR were selected by shifting the operating point to the right along the ROC curve, whereas thresholds favoring lower FPR were chosen by shifting to the left. The cut-off point was defined as the threshold that maximize the Youden index, calculated as the sum of TPR and 1-FPR which corresponds to true-negative ratio.^14^ We extracted several points at adequate intervals around the cut-off point along the ROC curve and defined the corresponding thresholds as candidate values. Using these threshold candidates, we experimentally constructed test OVLs with three ROIs generated from the PSPNet and YOLO outputs, utilizing original images from benign and multiple malignant specimens. The final optimized thresholds were selected based on the evaluation and expert insight provided by the most experienced pathologist reviewing the test OVLs. As a result, the optimal threshold for OVL1 and OVL2 was set at 0.899, whereas the threshold for OVL3 was set at 0.977, corresponding to the ROC cut-off point. The YOLO threshold to construct OVL2 was set at 0.8. Fig. 3 shows the ROC curve for PSPNet, with candidate and optimized thresholds indicated along the curve.

### Evaluation of CAD information

**Fig. 4:**
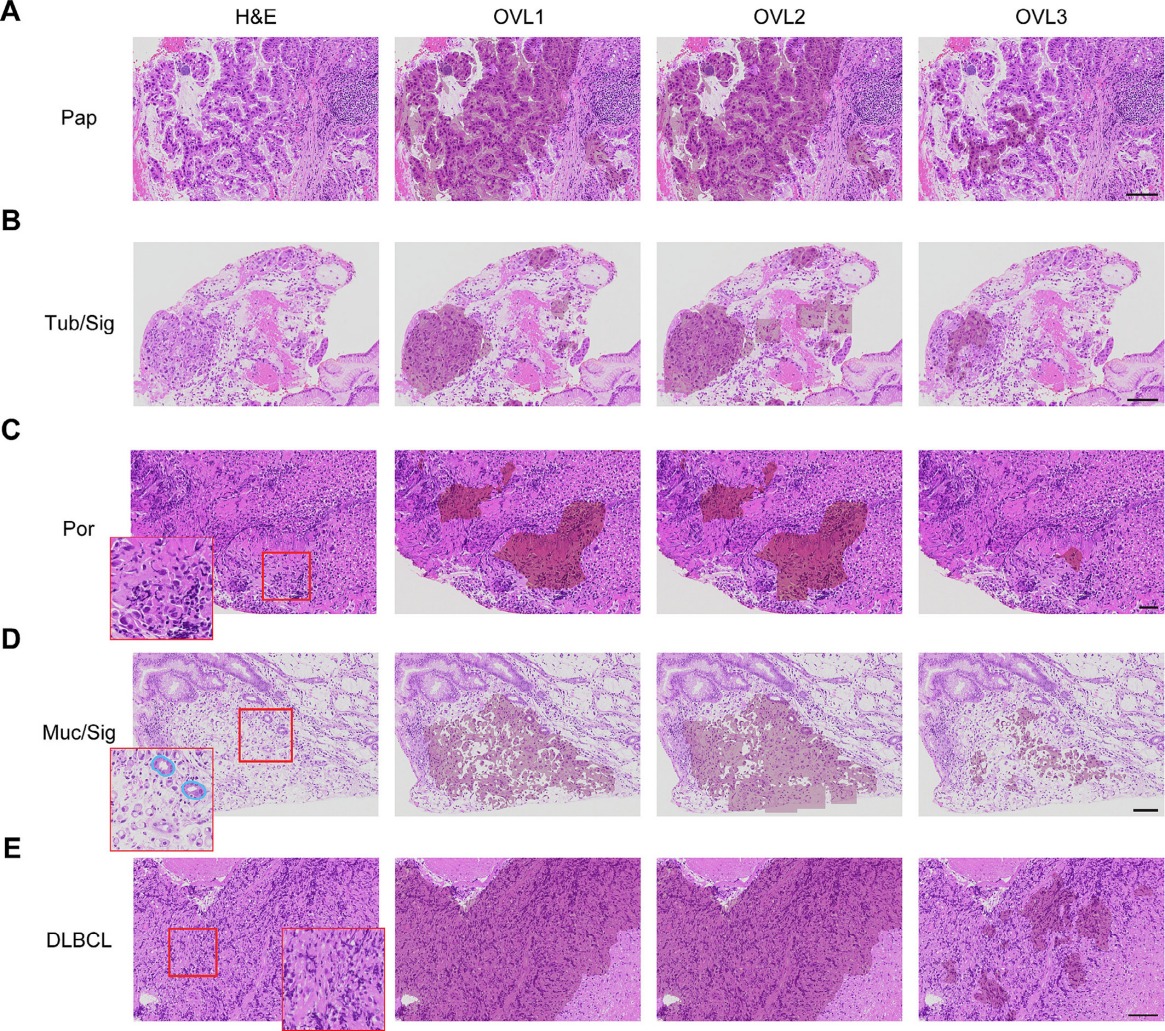
Representative image sets of original images and corresponding OVLs. (A) OVL1 and OVL2 identified majority of Pap regions, whereas OVL3 identified smaller regions. The regions identified by OVL1 and OVL2 contained mucinous areas surrounding adenocarcinoma cells. (B) OVL1 and OVL2 identified most Tub regions, and OVL2 further identified Sig regions as detected by YOLO algorithm (square regions). (C) OVL1 and OVL2 identified the same Por regions, as well as small areas of neutrophil aggregation. OVL3 identified a few adenocarcinoma cells of Por. (D) OVL1 and OVL2 identified the majority of Sig regions in Muc, with OVL2 delineating a broader area (square regions) compared to OVL1. OVL3 identified partial regions of Sig. OVL1 and OVL2 included benign glands (outlined in blue). (E) OVL1 and OVL2 identified majority of degenerated DLBCL regions, whereas OVL3 identified partial regions of degenerated DLBCL. Pap, papillary adenocarcinoma; Tub, Tubular adenocarcinoma; Sig, signet ring cell carcinoma; Por, poorly cohesive carcinoma; Muc, mucinous adenocarcinoma; DLBCL, diffuse large B-cell lymphoma. Scale bar, 50 μm. (For interpretation of the references to color in this figure legend, the reader is referred to the web version of this article.)

Next, we evaluated the performance of each OVL using 190 samples, comprising 92 Grade 1 (G1) samples and 98 Grade 5 (G5) samples. Number of samples used in the evaluation are shown in Table 1. 98 G5 samples included 13 well-differentiated Tub, 23 moderately differentiated Tub, 6 Pap, 4 solid type Por, 34 non-solid type Por, 10 Sig, 3 Muc, and 5 DLBCL. Samples were obtained from seventh hospital during January to July 2021. Fig. 4 shows representative image sets of original images and corresponding OVLs. The original H&E images comprise partial regions extracted from WSIs of malignant specimens representing various tumor types.

**Fig. 5:**
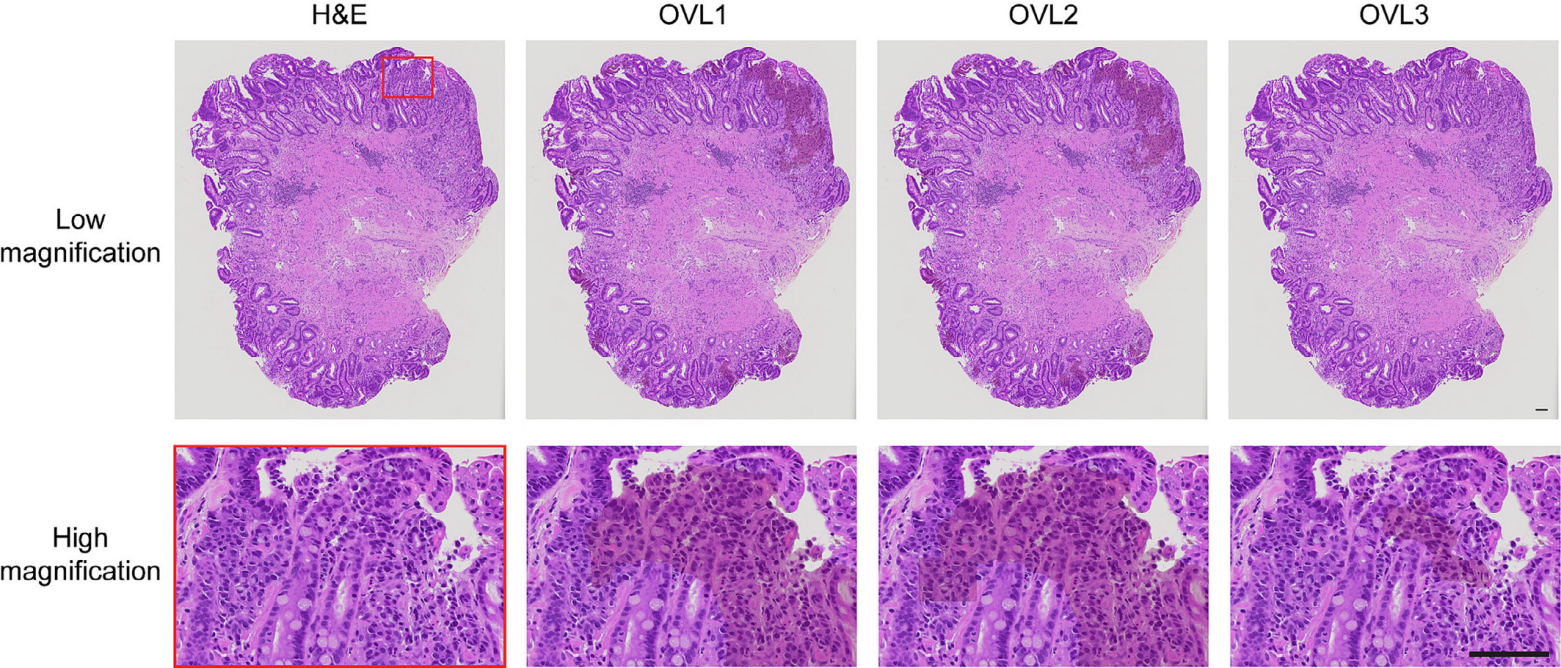
Representative original images and corresponding OVLs at low and high magnification. Case with Por. OVL1 and OVL2 identified the majority of Por regions, with OVL2 delineating a broader area (square regions) compared to OVL1. Regions detected by OVL3 were too small to be easily recognized at low magnification. Por, poorly cohesive carcinoma. Scale bar, 100 μm.

Overall, OVL1 and OVL2 detected majority of tumor regions, as both shared the same TPR and FPR thresholds in the ROC curve of PSPNet. OVL2 identified poorly cohesive carcinoma cells with greater sensitivity than OVL1, as determined by YOLO algorithm (Fig. 4B–D). At a higher threshold (i.e., higher specificity), region detected by OVL3 were smaller than those identified by the other OVLs; OVL3 did not detect non-malignant regions, whereas OVL1 and 2 included some benign structures, such as mucus (Fig. 4A), neutrophils (Fig. 4E), and benign glands (Fig. 4D). These findings align with our proposed approach: OVL1 and OVL2 are used to retrieve suspicious regions, whereas OVL3 supports definitive diagnosis. Next, the most experienced pathologist (KT) in the study group evaluated the performance of each OVL using the 190 samples with a WSI viewer. Table 2 presents the results of sensitivity, specificity, positive-predictive value (PV), and negative PV based on these 190 samples. We assessed the utility of each OVL at two different magnifications: low magnification for wide-field observation and high magnification for detailed zoom observation. Overall, OVL1 and OVL2 exhibited consistent sensitivity between wide-field and zoom observation. In contrast, zoom observation demonstrated higher sensitivity for OVL3, as the regions detected by OVL3 were too small to be recognized at low magnification in some cases (Fig. 5). Importantly, OVL3 demonstrated 100% specificity at both low and high magnification, indicating that a negative output from the system signifies the absence of malignancy. These findings highlight the potential utility of the system in routine clinical pathological practice.

**Table 2:** Evaluation of each OVL for 190 G1- and G5-gastric biopsy samples.

	Observation	Sensitivity (%)	Specificity (%)	Positive PV (%)	Negative PV (%)
OVL1	Wide-field	96.9	80.4	84.1	96.1
	Zoom	96.9	73.9	79.8	95.8
OVL2	Wide-field	98.0	78.3	82.8	97.3
	Zoom	98.0	79.7	78.0	97.0
OVL3	Wide-field	87.8	100	100	88.5
	Zoom	91.8	100	100	92.0

PV, predictive value.

### Trial test to evaluate clinical performance of AI-CAD

**Fig. 6:**
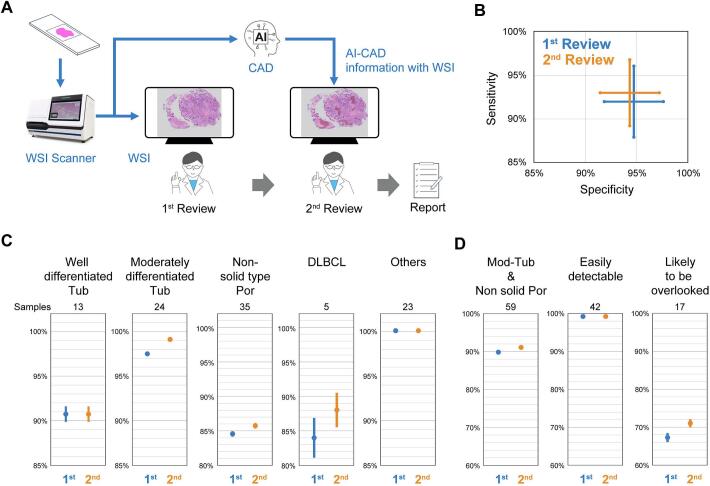
Performance of five board-certified pathologists with or without AI-CAD assistance. (A) Schematic diagram of the second reader workflow. CAD, computer-aided diagnosis. (B) Sensitivity and specificity from the first and second reviews, with error bars indicating 95% confidence intervals across five pathologists. (C) Sensitivities across histological subtypes from the first and second reviews. Others include Pap (6 samples), solid type Por (4), Sig (10), Muc (3). (D) Sensitivities for 42 easily detectable and 17 likely to be overlooked samples, comprising a total of 59 malignant tumors that included 24 moderately differentiated Tub and 35 non-solid type Por from panel C. Tub, tubular adenocarcinoma; Por, poorly cohesive carcinoma; DLBCL, diffuse large B-cell lymphoma; Sig, signet ring cell carcinoma; Muc, mucinous adenocarcinoma.

To assess the clinical performance of our AI-CAD system, we conducted a reader study in which pathologists reviewed cases with AI assistance. Specifically, to determine the utility of AI-CAD in supporting pathologists' detection of malignancy, we implemented an AI-supported diagnostic workflow based on the CADe concept, in which the AI-CAD system serves as the second reader (Fig. 6A). This type of CAD workflow system is commonly employed to enhance diagnostic performance by reducing missed malignancies and improving diagnostic confidence, and it is well recognized as a standard approach in the FDA guidance for the evaluation of CAD systems.^15^ In this workflow, each pathologist first recorded a group-level classification (G1–G5) without AI-CAD assistance (first review), followed immediately by a second classification with AI-CAD support (second review). In both reviews, pathologists made their diagnoses without clinical information. Group-level classification results from the first and second reviews were compared to evaluate the effectiveness of AI-CAD. 9 additional samples, comprising 7 G3 and 2 G4, were included in the above 190 samples (Table 1); 7 tubular adenoma with low-grade dysplasia, 1 suspicious for non-solid type Por, and 1 suspicious for moderately differentiated Tub. Malignant cases encompassed all tumor types used for training the CNN algorithms, excluding MALT lymphoma and GIST. The representation of each tumor type in the test set mirrored their relative incidence in clinical practice. For analytical purposes, grades G1–G3 were classified as benign and grades G4–G5 as malignant, in accordance with the aforementioned malignancy grading criteria (G1–G5).

Five board-certified pathologists participated in the study. Using a WSI viewer, they assessed the test images with standard zoom and pan operations. During the second review, they could toggle between the original images and each OVL at identical viewing coordinates. Before the test, the pathologists underwent a training session designed to guide effective interpretation of diagnosis generated by AI-CAD. This session introduced the conceptual basis and intended clinical utility of the OVLs, and included representative cases demonstrating typical ROI appearances, as presented by the most experienced pathologist. Through a structured pre-evaluation training session, we minimized potential recall and anchoring effects by instructing pathologists on the proper use of the AI-CAD system and by reviewing representative cases focusing on interpretation of OVLs.

After completion of the test, clinical performance was assessed by calculating the diagnostic sensitivity and specificity as statistical endpoints. To evaluate the significance of difference in these metrics with and without CAD support, we applied the general estimating equation (GEE),^16^ which accounts for correlations in multi-case and multi-reader studies. In this context, correlation arise from repeated reviews of the same test images (with and without AI-CAD) and from multiple pathologists interpreting the same images. As shown in Table 3 and Fig. 6B, both the overall diagnostic sensitivity and specificity across all pathologists exceeded 90%. Although the diagnostic sensitivity appeared higher with AI-CAD than without, wide 95% confidence intervals and *p*-value greater than 0.05 indicated that this difference was not statistically significant. To ensure statistical significance, as determined by Jackknife Alternative Free-response Receiver Operating Characteristic (JAFROC),^17^ we extended the study to more than 700 samples, assessed independently by 8 pathologists; validation of these findings is in progress. Furthermore, we examined the diagnostic sensitivity across histological subtypes to identify potential trends (Fig. 6C). Sensitivities for others including Pap, solid type Por, Sig, and Muc were 100% in both reviews, and false-negative cases were Tub, non-solid type Por, and DLBCL. For well-differentiated Tub and DLBCL, variability in pathological judgments among the participating pathologists may have contributed to differences in the assessment of malignancy: one pathologist diagnosed G5 well-differentiated Tub as G3 adenoma, whereas two pathologists diagnosed G4 degenerated DLBCL cells as G1 necrotic tumor cells.

**Table 3:** Overall sensitivity and specificity of the five pathologists.

	1st review Without AI-CAD			2nd review With AI-CAD			2nd–1st reviews With–without AI-CAD			
Sensitivity	Overall	95% CI-	95% CI+	Overall	95% CI-	95% CI+	Overall	95% CI-	95% CI+	P
	92.0 (%)	88.0 (%)	96.0 (%)	93.0 (%)	89.3 (%)	96.7 (%)	1.00 (%)	−0.16 (%)	2.16 (%)	0.091
Specificity	Overall	95% CI-	95% CI+	Overall	95% CI-	95% CI+	Overall	95% CI-	95% CI+	P
	94.7 (%)	91.9 (%)	97.6 (%)	94.3 (%)	91.5 (%)	97.2 (%)	−0.40 (%)	−1.19 (%)	0.38 (%)	0.315

CAD, computer-aided diagnosis; CIs, confidence intervals.

We further focused on moderately differentiated Tub and non-solid type Por by dividing them into two groups. Samples containing small or dispersed malignant foci, defined by malignant regions occupying less than 30% of the tissue, were categorized as likely to be overlooked (17 samples), whereas others were categorized as easily detectable (42 samples). The majority of false-negative cases were in the likely to be overlooked group. Importantly, second review showed improved diagnostic sensitivity in this group (Fig. 6D). We also evaluated the performance of each OVL, particularly in zoom observation, across the two categories (Supplementary Table S2). In the easily detectable category, OVL1, OVL2, and OVL3 correctly identified all samples as malignant (100% TPR). In the likely to be overlooked category, OVL3 showed a lower TPR than OVL1 and OVL2. Notably, although each OVL showed false-positive regions in both categories, those false-positive regions occupied less than 10% of the total tissue area in all samples.

## Discussion

The integration of AI model into digital pathology have been demonstrated to show high accuracy in various pathological diagnosis^18^, ^19^, ^20^, ^21^ in recent years. Previous reports on AI-based pathology systems generally follow two approaches: one relies on relatively small datasets, typically from a few to several dozen institutions, where pathologists make pixel-wise annotations for H&E-stained slides to apply supervised learning methods; the other leverages larger multi-institutional or publicly available collections of more than 1000 H&E slides using the reported diagnostic labels for weakly supervised learning methods.^22^, ^23^, ^24^, ^25^, ^26^, ^27^, ^28^, ^29^ Both approaches have advantages and limitations, but they share the fundamental principle of training AI systems on diagnoses rendered by pathologists. Because pathological diagnosis can vary due to subjective interpretation, training slides typically include representative malignant and benign morphologies with high inter-observer agreement, thereby yielding high diagnostic performance. Even under such optimized conditions, however, reported AI performance remains in the range of 85–95% sensitivity and specificity.^30^, ^31^, ^32^, ^33^ These settings, while useful for benchmarking, provide limited insight into the utility of AI systems in the complexity of real-world pathology practice.

To address this, we trained our system on diverse epithelial and non-epithelial tumors collected from six institutions and validated it using WSIs from an independent seventh institution. Importantly, the validation set included diagnostically challenging material, such as samples with minute malignant foci or malignant cells embedded within necrotic or degenerated tissue. Thus, our evaluation more closely reflects the conditions encountered in daily diagnostic practice.

Our AI system is based on the CADe concept and is designed to assist pathologists in identifying malignant regions within WSIs, which represents the initial and critical step in diagnosis, through supervised learning with precise pixel-wise annotation. A unique feature of our system lies in combining two independent algorithms (PSPNET and YOLO) and applying dual thresholding to PSPNET, which enabled us to define three operational validity levels (OVLs): (OVL1) PSPNET >0.899 alone, (OVL2) PSPNET >0.899 and YOLO >0.8, and (OVL3) PSPNET >0.977 alone. OVL1 achieved a sensitivity of 96.9%, OVL2 further increased it to 98% by detecting broader regions of signet-ring cell carcinoma, and OVL3 achieved a specificity of 100% (Table 2). On the other hand, OVL1 and OVL2 exhibited broader false-positive areas compared to OVL3, whereas OVL3 captured narrower true-positive regions (Fig. 4, Fig. 5). As a result, the system enabled AI-based detection of malignant regions already at the stage of low-magnification observation, thereby aligning with the practical workflow of pathologists. Indeed, pathologists utilizing these features demonstrated a modest but consistent trend toward improved diagnostic sensitivity (Fig. 6A, B and Table 3). This finding suggests that such an AI-assisted approach may enhance diagnostic accuracy and reduce workload in routine pathology.

Further analysis focusing on the histological subtypes indicated that cases of moderately differentiated Tub and non-solid type Por, especially which contain small, dispersed malignant foci, were associated with a higher incidence of false-negative diagnosis (Fig. 6D). These findings highlight a potential role for the system in enhancing diagnostic sensitivity, particularly in challenging cases, by converting false-negative into true-positive identifications. Although the false-positive regions occupied less than 10% of the total tissue area in all challenging cases (Table S2), it is essential for pathologists to distinguish between true- and false-positive findings and to make accurate judgments without uncritically accepting AI-based results. Importantly, diagnostic sensitivity showed a trend toward improvement when appropriate combinations of OVL1, OVL2, and OVL3 were applied, particularly for the *likely to be overlooked* (risky) samples and DLBCL cases (Fig. 6C and D). The present validation was limited by the number of WSIs included, and statistical significance was not achieved. To further substantiate these findings, additional validation using a larger dataset and assessments by eight pathologists are currently ongoing.

## CRediT authorship contribution statement

Concept and design: KT, DT, KK, CN, and SK. Search and collection of the samples and data: DT, KK, MM, NT, SM, MA, NK, TK, and KT. Experiment conduction: DT, KK, AS, AK, AS, RY, and KT. Analysis of data and interpretation: KT, DT, KK, CN, and SK. Statistical analysis: KT, CN, and SK. Manuscript writing and review: KT, DT, KK, CN, and SK.

## Ethics approval and consent to participate

This study was conducted in accordance with the Declaration of Helsinki and the Japanese Ethical Guidelines for Medical and Biological Research Involving Human Subjects. The requirement for informed consent was waived due to the retrospective design of the study. Participants were given the opportunity to decline inclusion in the study through opt-out notifications provided on each institution's website or through on-site postings within the hospital. Institutional review board approval for this study was obtained from all the institutions that participated in the study.

## Declaration of competing interest

The authors declare that they have no known competing financial interests or personal relationships that could have appeared to influence the work reported in this article.

## Data Availability

All data generated or analyzed during this study are included in this published article.

## Appendix A Supplementary data

Supplementary material 1 Training sample sets and the performance of OVLs Supplementary material 1
