# Supplementary material for: Development of an artificial intelligence-based computer-aided detection system for routine gastric biopsy diagnosis
Source: J Pathol Inform. 2026 Mar 19;21:100654. doi: 10.1016/j.jpi.2026.100654 (PMC13098442; doi:10.1016/j.jpi.2026.100654)
Supplement: Supplementary material 1 — Training sample sets and the performance of OVLs [file mmc1.docx]

**Supplementary tables**

Table S1. Number of WSIs and samples used for training

| System | Gastric biopsy specimen | No. WSIs | No. samples |
| --- | --- | --- | --- |
| PSPNet | Benign | 525 | 1,105 |
|  | Malignant tumor | 426 | 820 |
|  | Tubular adenocarcinoma (Tub) | 170 | 294 |
|  | Poorly cohesive adenocarcinoma (Por)  including signet ring cell type | 143 | 283 |
|  | Papillary adenocarcinoma (Pap) | 23 | 35 |
|  | Mucinous adenocarcinoma (Muc) | 2 | 3 |
|  | GIST | 17 | 30 |
|  | MALT | 49 | 107 |
|  | DLBCL | 22 | 68 |
| YOLO | Benign | 756 | 1,373 |
|  | Signet ring cell carcinoma (Sig) | 60 | 93 |

WSI, whole slide image. GIST, gastrointestinal stromal tumor. MALT, mucosa-associated lymphoid tissue. DLBCL, diffuse large B-cell lymphomas.

Table S2. Performance of each OVL in moderately differentiated Tub and non-solid type Por

| Categories | No. samples | Histology | | OVL1 | | OVL2 | | OVL3 | |
| --- | --- | --- | --- | --- | --- | --- | --- | --- | --- |
|  |  | Mod-Tub | Non-solid por | True positive | False positive | True positive | False positive | True positive | False positive |
| Easily  detectable | 42 | 21 | 21 | 42 (100%) | 16 (38.1%) | 42 (100%) | 16 (38.1%) | 42 (100%) | 6  (14.3%) |
| Likely  to be  overlooked | 17 | 14 [1] | 3 [1] | 14 (82.4%) | 7 (41.2%) | 15 (88.2%) | 7 (41.2%) | 8 (47.1%) | 0  (0.0%) |
| Total | 59 | 35 [1] | 24 [1] | 56 (94.9%) | 23 (39.0%) | 57 (96.6%) | 23 (39%) | 50 (84.7%) | 6  (10.2%) |

Numbers in brackets [ ] indicate G4 samples. Samples with malignant regions occupying less than 30% of the tissue were categorized as likely to be overlooked. In false positive cases, the false positive regions occupied less than 10% of the total tissue area. Mod-Tub: moderately differentiated tubular adenocarcinoma. Por, poorly cohesive carcinoma.
